# Evolution of the Selfing Syndrome in *Arabis alpina* (Brassicaceae)

**DOI:** 10.1371/journal.pone.0126618

**Published:** 2015-06-03

**Authors:** Andrew Tedder, Samuel Carleial, Martyna Gołębiewska, Christian Kappel, Kentaro K. Shimizu, Marc Stift

**Affiliations:** 1 Institute of Evolutionary Biology and Environmental studies, University of Zurich, Zurich, Switzerland; 2 Ecology, Department of Biology, University of Konstanz, Konstanz, Germany; 3 Institut für Biochemie und Biologie, Universität Potsdam, Potsdam-Golm, Germany; Chiba University, JAPAN

## Abstract

**Introduction:**

The transition from cross-fertilisation (outcrossing) to self-fertilisation (selfing) frequently coincides with changes towards a floral morphology that optimises self-pollination, the selfing syndrome. Population genetic studies have reported the existence of both outcrossing and selfing populations in *Arabis alpina* (Brassicaceae), which is an emerging model species for studying the molecular basis of perenniality and local adaptation. It is unknown whether its selfing populations have evolved a selfing syndrome.

**Methods:**

Using macro-photography, microscopy and automated cell counting, we compared floral syndromes (size, herkogamy, pollen and ovule numbers) between three outcrossing populations from the Apuan Alps and three selfing populations from the Western and Central Alps (Maritime Alps and Dolomites). In addition, we genotyped the plants for 12 microsatellite loci to confirm previous measures of diversity and inbreeding coefficients based on allozymes, and performed Bayesian clustering.

**Results and Discussion:**

Plants from the three selfing populations had markedly smaller flowers, less herkogamy and lower pollen production than plants from the three outcrossing populations, whereas pistil length and ovule number have remained constant. Compared to allozymes, microsatellite variation was higher, but revealed similar patterns of low diversity and high Fis in selfing populations. Bayesian clustering revealed two clusters. The first cluster contained the three outcrossing populations from the Apuan Alps, the second contained the three selfing populations from the Maritime Alps and Dolomites.

**Conclusion:**

We conclude that in comparison to three outcrossing populations, three populations with high selfing rates are characterised by a flower morphology that is closer to the selfing syndrome. The presence of outcrossing and selfing floral syndromes within a single species will facilitate unravelling the genetic basis of the selfing syndrome, and addressing which selective forces drive its evolution.

## Introduction

Most angiosperms are outcrossing, but transitions to self-fertilisation (selfing) are frequent [1,2]. Based on phylogenetic data, these transitions appear to be unidirectional and irreversible, with selfing as the derived state [3–6]. The repeated parallel transition to selfing has provided a unique opportunity to find recurrent patterns in adaptive evolution among different species [7,8]. Selfing provides two distinct advantages when compared to outcrossing [1,2,9]: transmission advantage and reproductive assurance. Selfers have a transmission advantage because they pass their complete genome to the next generation without dilution of their genetic material from a mating partner. At the same time, they can also act as outcross pollen donors for seed produced by other individuals [10]. Reproductive assurance is due to selfers being able to reproduce when mates are limited or when pollinators are scarce [11]. For selfing to evolve from an outcrossing background, any barriers to self-fertilisation (e.g., self-incompatibility mechanisms) must break down, and transmission advantage and reproductive assurance must outweigh inbreeding depression and any other negative effects of inbreeding [12,13]. Conditions conducive to the evolution of selfing are for example found during postglacial geographic range expansion, where mate and pollinator limitation are likely [14,15].

Transitions to selfing often result in speciation in which changes in the floral syndrome play an important part [16,17]. Selfing species have a typical floral syndrome termed the selfing syndrome [18]. Compared with outcrossing syndromes, the selfing syndrome consists of smaller flowers that open less, have reduced herkogamy (a shorter distance between stigma and anthers) and a tendency towards reduced pollen: ovule ratios, nectar production and scent emission [18]. Several selective forces may drive the evolution of the selfing syndrome, for example selection for rapid maturation in marginal habitats leading to a reduction in overall organ size [19], selection for smaller flowers through preferential predation on larger flowers by florivores [20,21], or selection for increased resource allocation to progeny and reduced allocation to attractive tissues through the reduced requirement for pollinator attraction [22]. The abundant examples of closely related self-incompatible and self-compatible taxa with contrasting floral syndromes (for example *Ipomoea cordatotriloba* and *I*. *lacunosa* [23]; or *Capsella grandiflora* and *C*. *rubella* [16]) suggest that the evolution of selfing is a major force driving speciation in angiosperms. Accordingly, the evolutionary transition from outcrossing, self-incompatible ancestors to self-compatible, selfing lineages is of prime interest to evolutionary botanists [5].

Comparisons between closely related taxa with contrasting mating systems have been used to start unravelling the genetic basis of the loss of self-incompatibility and of floral changes towards the selfing syndrome. Interspecific crosses between outcrossing and selfing sister taxa have been used for linkage mapping of the selfing syndrome in a limited number of systems including *Capsella* [24], *Leptosiphon* [25], *Lycopersicon* [26,27] and *Mimulus* [28,29]. The common pattern emerging from these studies is that a large number of loci contribute to the floral traits that differ between the outcrossing and selfing sister species. Specifically in *C*. *rubella* it has been suggested that the selfing syndrome has evolved from the common ancestor with *C*. *grandiflora* in a stepwise manner, involving multiple consecutive mutations at different loci [24]. Further unravelling the timeline of such stepwise evolution requires systems in which outcrossing and selfing lineages have diverged less than the 30-50ky that has been estimated for *C*. *grandiflora* and *C*. *rubella* [16,30]. Therefore, it is of interest to characterise systems with intraspecific mating system and floral syndrome variation, which has the extra advantage that these may be crossed more readily. Such systems would also facilitate more directly addressing the selective forces that drive the evolution of the selfing syndrome.

The natural variation in mating system that has been discovered in *Arabis alpina* may provide a promising model in this context. Across its European range, *A*. *alpina* occurs in alpine and sub-alpine areas from Spain to Scandinavia [31,32]. This wide distribution range has been attributed to recent expansion after glacial periods [31]. In its northern range, *A*. *alpina* is self-compatible with variable *F*_IS_ values suggesting a mainly selfing reproductive strategy, with a maintained ability to outcross [33–35]. In three populations, allozyme genotyping of progeny arrays had confirmed that plants almost exclusively reproduced by selfing (outcrossing rates <0.15 [34]). South of the Alps, *F*_IS_ values suggested a predominantly outcrossing strategy [33] and in line with this we recently discovered three self-incompatible populations in the Apuan Alps with outcrossing rates >0.78 and high polymorphism in the self-incompatibility gene *SRK* [34].

With its draft genome assembly publicly available [36], *A*. *alpina* is an emerging model species, among others for studying perenniality [37] and local adaptation [38,39]. Using transgenic techniques and mutant screenings, the molecular pathways responsible for perennial flowering have been extensively studied [40–42]. In addition, given the presence of both self-incompatible outcrossing populations and predominantly selfing populations, *A*. *alpina* may be a feasible model to study the molecular and evolutionary basis of the transition to selfing, and the floral changes associated with this transition. However, it is still unknown whether changes towards a selfing syndrome have occurred in *A*. *alpina*.

**Fig 1:**
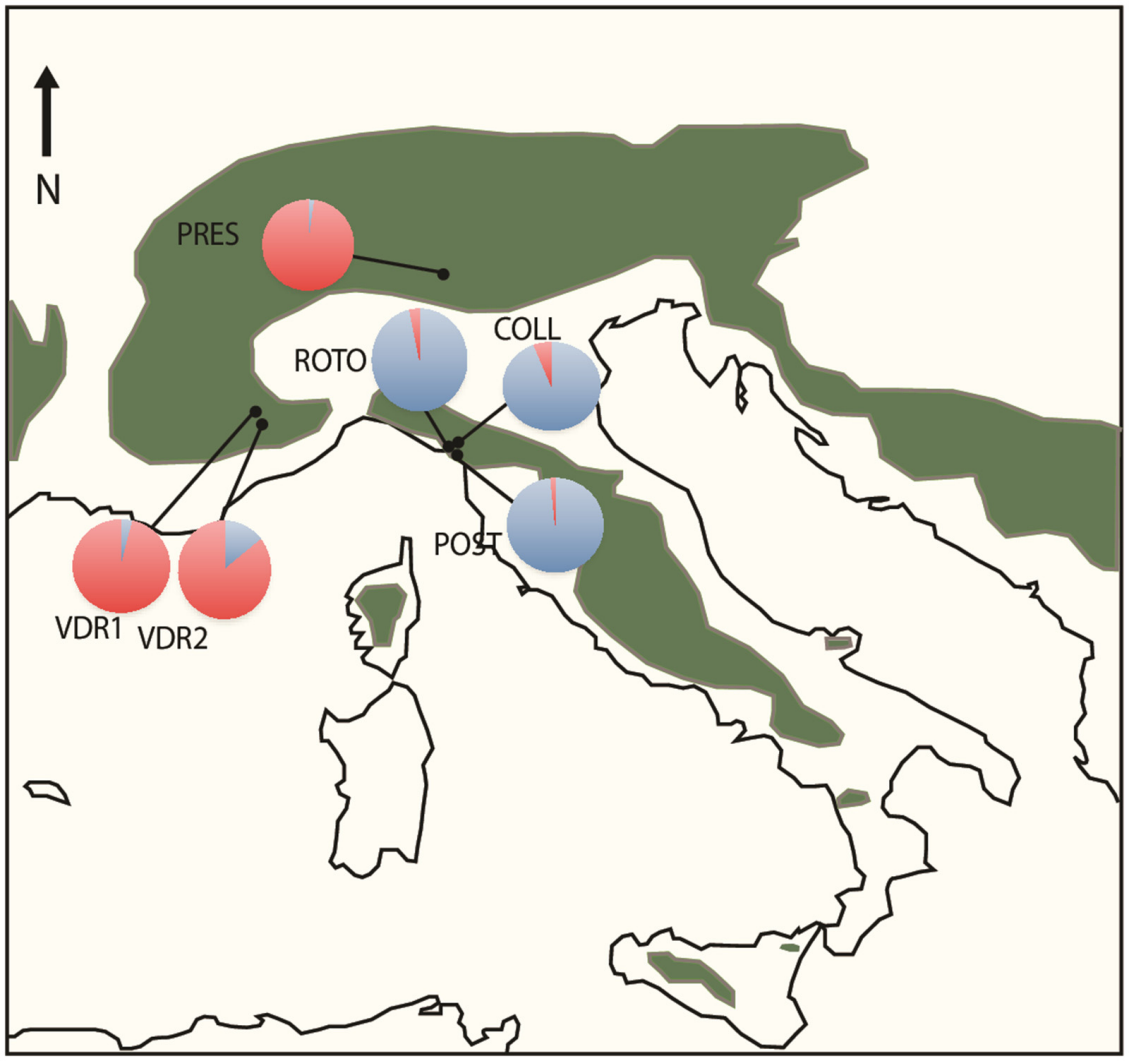
Sampling locations of three outcrossing populations and three selfing populations of *Arabis alpina* (details on outcrossing rates, see Table 3). Pie charts indicate the population mean posterior assignment probability based on Bayesian clustering analysis (Fig 5). The predominantly blue cluster includes the three outcrossing populations: COLL = Colli; POST = Porte Strazzema; ROTO = Rotondo. The predominantly red cluster includes the three selfing populations: PRES = Presolana; VDR1 and 2 = Val de Roya 1 and 2.

In this paper, we use six populations (Fig 1) for which outcrossing rates had been estimated previously to test whether plants in selfing populations (Maritime Alps and Dolomites) are associated with smaller flowers compared to outcrossing populations (Apuan Alps), and whether other traits (herkogamy, pollen and ovule number) have also changed towards states typical for the selfing syndrome. In addition, we used a set of microsatellite markers [35] to confirm previous population genetic estimations (allelic richness, Ho, He, Fis, population structure) based on allozymes, because these allozymes had suffered from lack of variation in the selfing populations [33,34].

## Materials and Methods

### Plant material

To compare floral morphology (size and herkogamy) among three outcrossing and three selfing populations of *Arabis alpina* (Fig 1), we made use of a collection of randomly arranged individually potted plants growing in a common garden environment (Botanical Garden, University of Konstanz) since October 2012. These individuals started to flower in April 2013, and again in March 2014. The plants originated from wild-collected maternal seed families from three outcrossing populations in the Apuan Alps in Italy (Rotondo, 14 seed families; Colli, 14 seed families; Porte Strazzema, 13 seed families) and three selfing populations, one from the Dolomitic Alps in Italy (Presolana, 13 seed families) and two from the Maritime Alps in France (Val de Roya 1, 14 seed families; Val de Roya 2, 4 seed families) [34] (Fig 1). These were sown in October 2011 for another experiment. Each seed family was represented by a single plant, so the total design included 41 individuals from outcrossing, and 31 individuals from selfing populations.

### Flower macrophotography

To determine flower size (petal area) and herkogamy, in spring 2013, we took standardised photographs of flowers using a Nikon D7100 DSLR camera with a Sigma 150mm F2.8 EX DG OS HSM APO Macro lens that was mounted on a height-adjustable support. For each plant, we photographed two to five flowers with the camera at the minimum focal distance. The developmental state of flowers was standardised by collecting flowers between 1000 h and 1600 h, when mature flowers were fully opened and only using flowers of which the anthers were in the process of dehiscing, and that still had bright white petals (i.e., petals that were neither losing brightness, nor had already started wilting; petals tend to lose brightness 1–3 days after anther dehiscence). If an inflorescence had multiple flowers, these criteria normally meant that we used the youngest fully opened flower. Immediately after collection, we photographed flowers against a black background (1% agar with 0.5% activated carbon), always including a size standard in the frame. First, to record petal area, we placed the pedicel of intact flowers in a capillary tube, and positioned the top-view plane of the flower parallel to the lens plane. Then, to record herkogamy, we carefully removed the petals and two of the long stamens, and positioned the plane through the style and short stamens parallel to the lens plane.

### Flower size, herkogamy and shape analysis

**Fig 2:**
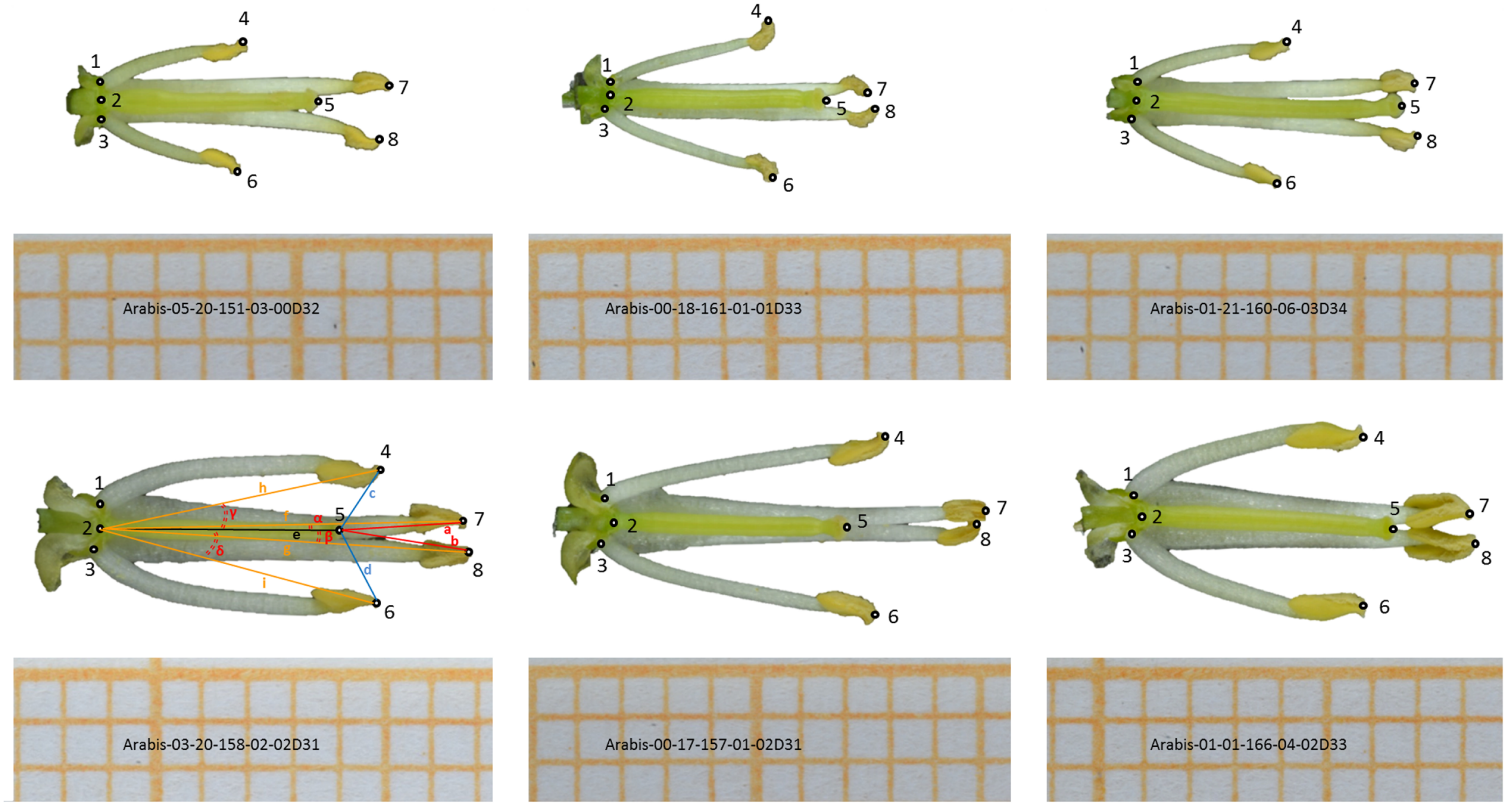
Placement of eight landmarks on dissected side view photographs of *Arabis alpina* flowers. Photos are representative examples for each of the three selfing populations (top panel) and the three outcrossing populations (bottom panel). Numbers indicate landmarks: 1—base of short left stamen; 2—base of pistil; 3—base of short right stamen; 4—anther tip of short left stamen; 5—stigma tip; 6—anther tip of short right stamen; 7—anther tip of long left left stamen; 8—anther tip of long right stamen. Within one of the pictures, lines indicate the measure taken based on the landmarks: average herkogamy long stamens (red lines) as *(a*+*b*)/2; average herkogamy short stamens (blue lines) as (*c*+*d*)/2; average length long stamens (orange lines) as *(f*+*g*)/2; average length short stamens (orange lines) as (*h*+*i*)/2; average angle long stamens (red double dashed line) as (*α*+*β*)/2; average angle short stamens (red double dashed line) as (*γ*+*δ*)/2.

Flowers and size standard were separated (segmented) from the black background using Matlab R2014a (Version 8.3) and its image processing toolbox by applying Otsu's thresholding method. Area and perimeter for both objects (flower and standard) were extracted using the *regionprops* function, automatically identifying the objects based on their inherently different shapes (standards were rectangular and thus had a higher eccentricity than the flowers). Flower parameters were scaled using information from the size standard. Flower shapes (i.e., the shapes of the petal outlines) were described by calculating a dissection index as $\frac{perimeter}{\sqrt{area}}$. To quantify herkogamy on photographs of the dissected flowers, we used the software TPSdig2 (version2, http://life.bio.sunysb.edu/morph/) to place 8 digital landmarks on the dissected flower (Fig 2). Landmark coordinates were imported in R (version 2.15.3 [43]), and using basic trigonometric functions we calculated mean herkogamy and angle for both short and long stamens and the ratio between the short and long stamen length (Fig 1 and Table 1).

**Table 1:** Overview of landmark-based traits extracted from dissected side view photographs (see Fig 2 for an overview of the landmarks).

Trait	Formula^a^
**Mean herkogamy long stamens (mm)**	$\frac{d(L5-L7)+d(L5-L8)}{2}$
**Mean herkogamy short stamens (mm)**	$\frac{d\left(L5-L4\right)+d\left(L5-L6\right)}{2}$
Mean angle long stamens (degrees)	$\frac{∠\;L7L2L5+∠\;L8L2L5}{2}$
Mean angle short stamens (degrees)	$\frac{∠\;L4L2L5+∠\;L6L2L5}{2}$
**Pistil length (mm)**	***d(L2–L5)***
Mean long stamen length	$\frac{d(L2-L7)+d(L2-L8)}{2}$
Mean short stamen length	$\frac{d(L2-L4)+d(L2-L6)}{2}$

Traits for which the analysis is presented in the main paper are in bold, analyses for the remaining traits are presented in S1 Table and S1 Fig.

^a^ d = distance (in mm)

### Pollen counting

To obtain pollen counts, in spring 2014, we collected three to six flower buds for four to eight plants per population (n = 5 for Rotondo, n = 7 for Porte Strazzema, n = 7 for Colli, n = 6 for Presolana, n = 8 for Val de Roya 1, n = 4 for Val de Roya 2). To standardise the developmental stage of buds, we only collected buds on which the petals were just starting to become visible (no more than 1–2 mm) between 1000 h and 1600 h. Buds were manually opened and dried overnight at 65°C. To release pollen from the anthers, we added 100 μl of 5% Tween-20 and sonicated (Bioruptor Plus, Diagenode, Liege, Belgium) for 10 cycles of 30 seconds ‘ON’ and 30 seconds ‘OFF’. Finally we added 10 mL of CASY Tone buffer (Roche Diagnostics, Mannheim, Germany) and the total volume was then analysed on a CASY Model TT—Cell Counter and Analyzer (Roche Diagnostics, Mannheim, Germany), using the 150μm capillary (which covers a size range of 3.2–120 μm) with a measurement range from 12.5 to 25 μm. If aborted pollen was detected, the measurement range was altered to 7.5–25 μm to ensure accurate counting of both pollen types. Pollen counts were well below 10000 in 18 out of 137 cases (8 from outcrossing populations, 10 from selfing populations). As we assumed that these were due to technical artefacts, we excluded them from analysis (excluded samples had pollen counts that ranged from 269 to 7612, included samples ranged from 10100–218100).

### Ovule counting and ovary length measurement

To count ovules, we collected three to six buds in the same stage and from the same plants as used for pollen counting (for Rotondo, samples for two extra plants were included), and fixed them in ethanol: acetic acid (9: 1) for a minimum of 12 hours. To soften the tissue, pistils were cut at the receptacle and stigma, and soaked in 1 N NaOH for 2 h at 60°C. After softening, samples were placed onto a microscopic slide and ovule number was counted under a Leica DM5000 B microscope with a DIC prism (Leica, Wetzlar, Germany).

To determine ovary length on the same samples for which ovules had been counted, we took photographs with a Leica Monochrome Digital Camera DFC345 FX (Leica, Wetzlar, Germany) connected to the microscope (magnification 5x) using the Leica Application Suite AF version 1.0.0 build 0 (Leica, Wetzlar, Germany). To fit the whole ovary, 1–4 photographs were taken for each sample, with sufficient overlap to allow later merging of the images with the ‘photomerge’ function in Adobe Photoshop CC version 14.2.1 x 64 (Adobe Systems, California, USA). Using the merged images, we manually measured ovary length using ImageJ 1.48v.

### Statistical analysis

To test whether floral traits differed between mating systems, we used linear mixed models as implemented in the *lme* function in the *nlme* package in R (v2.15.3 [43]). The fixed part of the model was Mating System (outcrossing vs. selfing), and the random part of the model included Population (nested in mating system, since we compared three outcrossing and three selfing populations) and Plant (nested in Population, since multiple flowers were analysed for each plant). We inspected model residual structure for normality and variance homogeneity, and applied transformations to meet model assumptions where needed. With one exception, in such cases either a square root or log_e_ transformation was sufficient. In the case of pollen number there was strong variance heterogeneity among mating systems, which we addressed by modelling a separate variance for the factor levels outcrossing and selfing (using the subcommand *VarIdent* within the *lme* function), so that interpretation of results is not biased by variance heterogeneity [44].

### DNA extraction and microsatellite genotyping

For genetic analysis, in October 2012, we collected and dried (on silica) leaf tissue from plants from the same three outcrossing and selfing populations that we had used to study flower morphology. The genetic analysis included all 72 individuals for which flower morphology had been determined, and 23 additional samples for which tissue had been collected, but that had either died before the flower measurements (in winter 2012–2013) or that did not have flowers during the flower photography (in spring 2013). Sample sizes per population were: Rotondo (n = 23), Colli (n = 17), Porte Strazzema (n = 20), Val de Roya 1 (n = 15); Val de Roya 2 (n = 4), Presolana, n = 16). To extract DNA, up to 50 mg of desiccated tissue was ground for two 30-second bursts using a TissueLyser mixer-mill disrupter (Qiagen, CA, USA) with the addition of a single 5mm stainless steel bead per sample. Total genomic DNA was then extracted using a Biosprint 96 workstation (Qiagen, CA), following the manufacturers’ default protocols.

To determine whether populations clustered according to mating system and region of origin, we genotyped 12 microsatellite loci developed for *A*. *alpina* across all 96 individuals: DJ5E, DEET, 4MDH, A1T8T, 5GTC, 6U3S, A93Q, A4JW7, 9VSH, 7PJQ, BWF1 and 3Q19 [35]. In each case, the forward primer was tagged with one of four fluorescent dyes, 6-FAM, ATTO550, ATTO565, or Yakima yellow (MicroSynth AG, Balgach, St. Gallen, Switzerland). Products were amplified by singleplex PCR using the following reagents: 1 μL of 50–100 ng/μL DNA template, 4 μL 5x PCR buffer, 1 μL of 2 mM MgCl_2_, 1 μL of 10mM dNTPs, 0.2 μL of 10μM forward and reverse primer, 1U Taq polymerase, and ddH_2_O to a reaction volume of 20 μL. Thermocycling was performed on PTC-200 (MJ research, Watertown, MA, USA) machines using the following programme: initial denaturation at 94°C for 15 min followed by 30 cycles of 94°C for 30 s, 57°C for 90 s, 72°C for 60 s, (ramp to 72°C at 0.7°C/s) and a final 72°C extension for 30 min. PCR products (1:200 dilutions) were genotyped using an ABI 3730 sequencer. Genotypes were analyzed using GENEMAPPER 4.0 (Applied Biosystems, Foster City, CA, USA) and corrected manually.

To confirm previous findings based on allozymes [33], for each population we used our microsatellite genotypes to calculate the average number of alleles per locus, expected (*H*_E_) and observed (*H*_O_) heterozygosity, and the inbreeding coefficient (*F*_IS_) using GENEPOP 4.0.10 [45,46].

### Bayesian inference of population structure

To analyse clustering of populations in relation to mating system and geography, we used the Bayesian clustering algorithm implemented in STRUCTURE 2.3.4 [47]. This method uses a multilocus genotype to probabilistically assign individuals to one or more clusters. Using the admixture model with default settings (correlated allele frequencies), we ran 10 simulations per prior *K* (*K* = 1 to *K* = 6); for a burn-in period of 200,000 generations and 1,000,000 MCMC replicates after burn-in. We plotted *K* vs. the likelihoods obtained over all simulations and inferred the optimal number of clusters *K* as recommended by the STRUCTURE manual. Additionally, we confirmed *K* based on the ΔK method [48], using STRUCTURE HARVESTER v0.46 [49] to calculate the mode of second order derivatives of the likelihood distribution divided by the standard deviation *s* across replicates (*m|L′′(K)|/s[L(K)])*, which was plotted against *K* for visual evaluation. CLUMPP 1.1 was used to combine simulation output for each *K* [50].

## Results

### Floral changes towards the selfing syndrome

**Fig 3:**
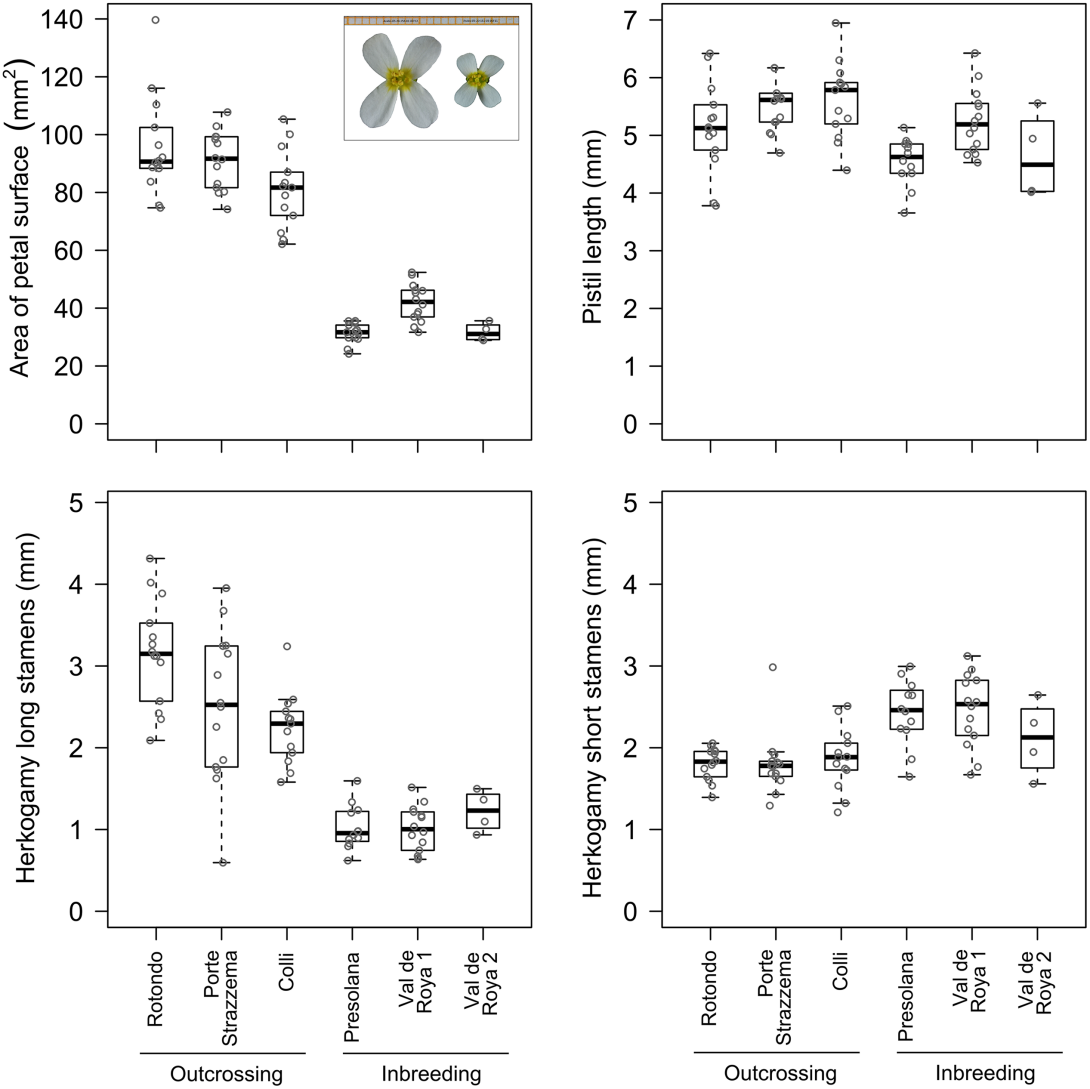
Boxplots for mature flower traits of three outcrossing and three selfing populations of *Arabis alpina*. A) petal area of landing platform, inlay shows two representative examples of the original photographs; B) pistil length; C) absolute herkogamy of long stamens; D) absolute herkogamy of short stamens. The individual points indicate the mean trait values for the replicate flowers analysed within individual plants on which the boxplots were based.

**Fig 4:**
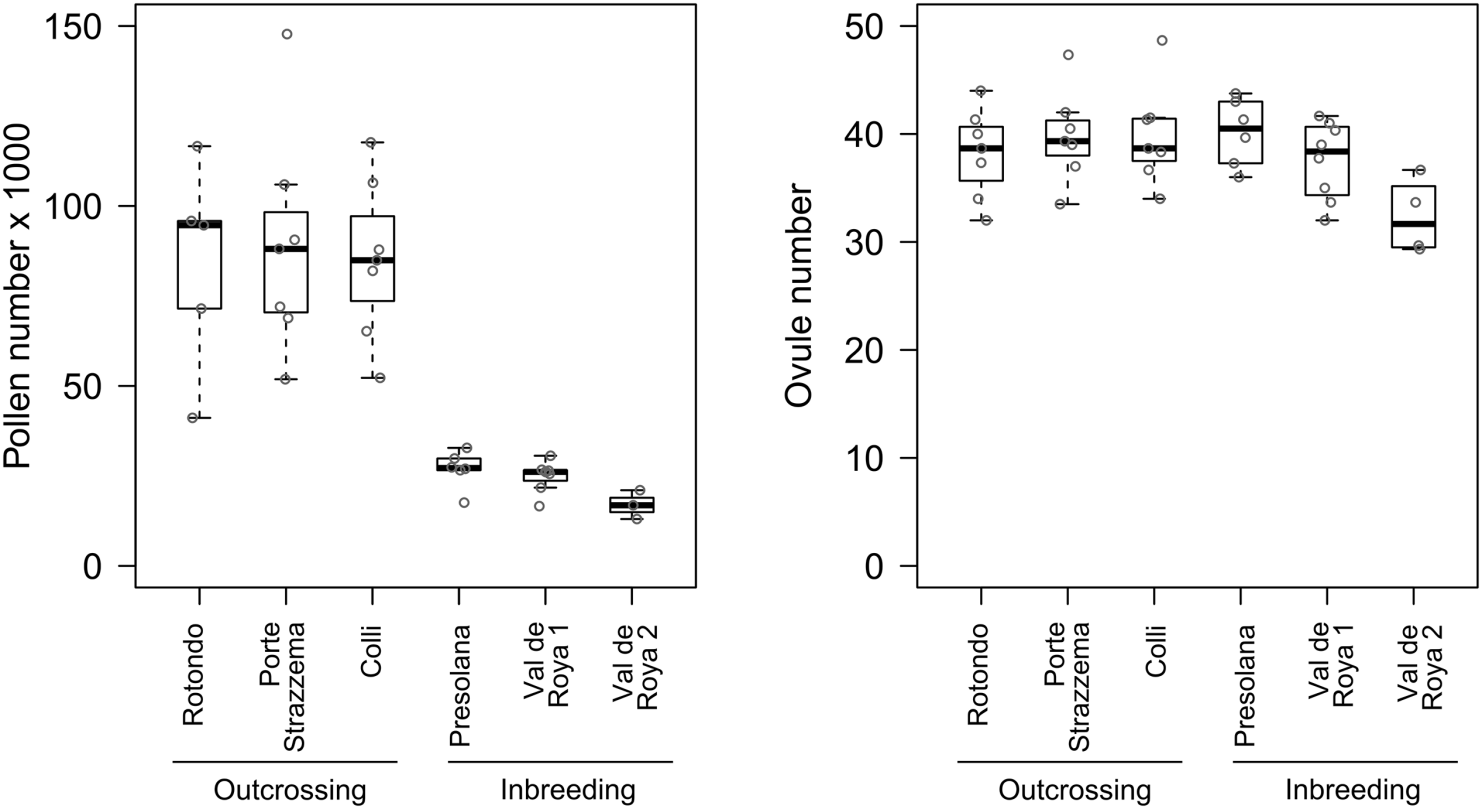
Gamete production in three outcrossing and three selfing populations of *Arabis alpina*. A) Pollen numbers; (B) and ovule numbers. Individual points indicate the mean trait values for the replicate flowers analysed within individual plants on which the boxplots were based.

Compared to outcrossing populations, plants from selfing populations had 2.5 times smaller flowers (petal area), a 2.5 fold reduction in the degree of herkogamy of the long stamens, and a 1.3 fold increase in herkogamy of the short stamens (Fig 3 and Table 2). Moreover, selfing populations produced 3.4 times fewer pollen grains while the number of ovules was not significantly different (Fig 4 and Table 2). Using the mean pollen number and mean ovule number of each plant, this meant that pollen: ovule ratios were markedly lower in the selfing populations (667, 669 and 546, for Presolana, Val de Roya 1 and Val de Roya 2, respectively) than the outcrossing populations (2187, 2244 and 2188 for Rotondo, Porte Strazzema and Colli, respectively). Pollen sizes were slightly larger for the selfing populations (S1 Table).

**Table 2:** Linear mixed model analysis of the effect of Mating System on floral traits of *Arabis alpina*.

Trait (unit of measurement)	Transformation for analysis	Model estimate (transformed) of difference between outcrossing and selfing means^a^	t-value	p (df = 4)^b^
Flower area (mm^2^)	log_e_	**-0.94**	**-8.68**	**0.001**
Pistil length (mm)	square root	-0.14	-2.15	0.0978
Herkogamy short stamens (mm)	-	**+0.58**	**6.54**	**0.0028**
Herkogamy long stamens (mm)	square root	**-0.58**	**-5.94**	**0.004**
Pollen number [x1000] (count)^c^	-	**-61.6**	**-10.7**	**<0.001**
Ovule number (count)	-	-2.55	-1.20	0.298

Cases where model estimates for selfing populations differ significantly from those of the outcrossing populations are indicated in bold.

^a^ Model Fixed part: Mating system; Random part: Population (nested in Mating System) and Plant_ID (nested in Population), analysed with the *lme* function in the *nlme* package in R [43]

^b^ df: degrees of freedom. For testing differences between mating system df = 4 because there were three outcrossing and three selfing populations

^c^ This model included a separate variance for the two mating systems (varIdent option of lme), as there was a large variance heterogeneity among mating systems that could not be removed by transformation.

The reduced herkogamy of the long stamens in selfing populations could not be attributed to an increased angle between pistil and long stamens. In fact the angle was larger for selfing populations (S1 Table), which would have had the opposite effect (i.e., increasing herkogamy). Pistil length did not differ between outcrossing and selfing populations (Fig 3 and Table 2), and hence the reduced herkogamy of the long stamens (Fig 3 and Table 2) was mainly due to a reduced long stamen length (and anther size) in selfing populations (S1 Table). The increased herkogamy of the short stamens (Fig 3 and Table 2) was due to a combined effect of an increased angle between pistil and short stamens, and a reduced short stamen length in selfing populations (S1 Table). There were no flower shape differences based on dissection index (S1 Table).

### Diversity indices and Bayesian inference of population structure

**Fig 5:**
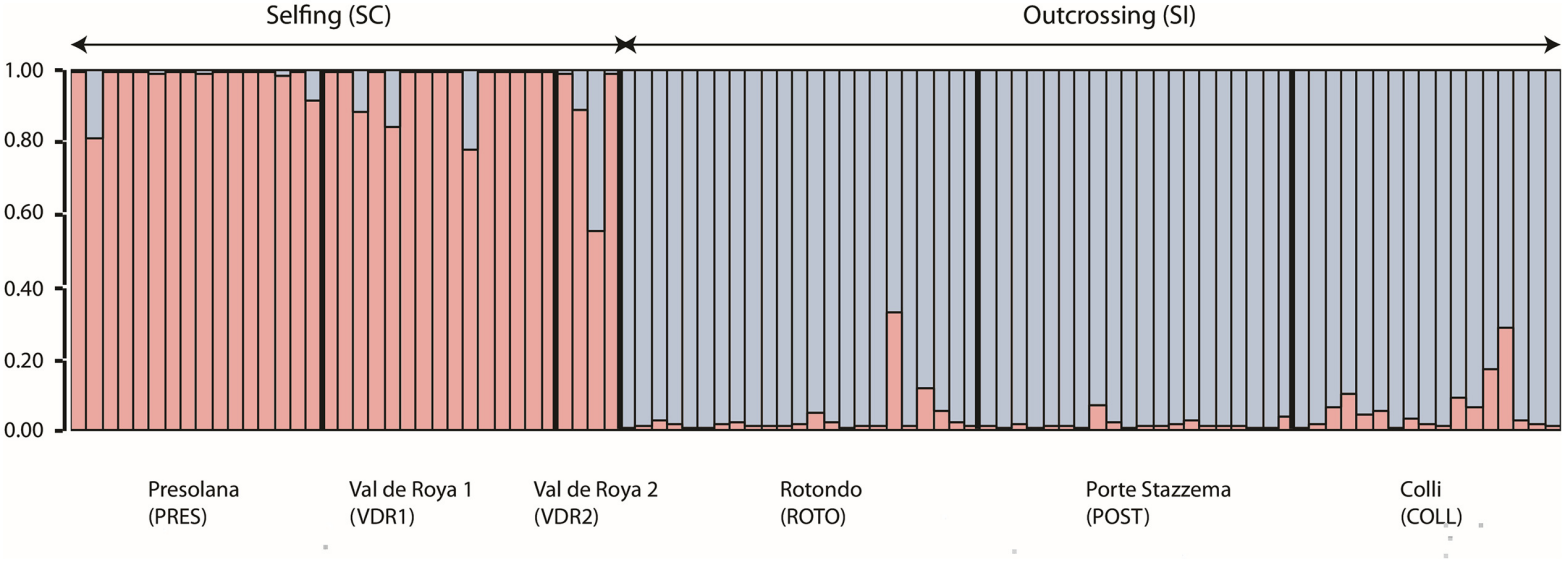
Bayesian clustering (STRUCTURE) based on 12 microsatellite loci. Individual posterior probabilities for inferred number of clusters (k = 2, see S1 Fig for justification). Red and blue bars indicate the individual posterior probability of belonging to cluster 1 and 2, respectively.

For the microsatellite loci, inbreeding coefficients were consistently higher for the selfing populations and genetic diversity was lower (Table 3). Both visual interpretation of the STRUCTURE output and the delta K method indicated optimal clustering at K = 2 (S1 Fig). These clusters corresponded with mating strategy and region of origin, thus separating the selfing populations (Presolana, Val de Roya 1 and 2) into the first cluster, and the outcrossing populations (Rotondo, Porte Strazzema and Colli) into the second cluster (Fig 5).

**Table 3:** Population genetic summary statistics per microsatellite locus for outcrossing and selfing populations.

Outcrossing populations												
Locus	Rotondo (*T*_*m*_ = 0.98)				Porte Strazzema (*T*_*m*_ = 0.89)				Colli (*T*_*m*_ = 0.78)			
	*N*_A_	*H*_O_	*H*_E_	*F*_IS_	*N*_A_	*H*_O_	*H*_E_	*F*_IS_	*N*_A_	*H*_O_	*H*_E_	*F*_IS_
DJ5E	3 (0)	0.43	0.61	0.30	4 (1)	0.55	0.57	0.04	1 (0)	0.00	0.00	-
DEET	4 (0)	0.83	0.67	-0.25	6 (2)	0.85	0.78	-0.09	4 (1)	0.67	0.66	-0.01
4MDH	8 (3)	0.64	0.84	0.24	7 (4)	0.55	0.70	0.22	4 (0)	0.88	0.74	-0.20
A1T8T	5 (1)	0.53	0.73	0.27	5 (0)	0.63	0.72	0.12	3 (1)	0.50	0.56	0.10
5GTC	6 (3)	0.35	0.53	0.35	4 (0)	0.45	0.64	0.30	5 (3)	0.59	0.74	0.20
A93Q	4 (1)	0.30	0.53	0.42	3 (0)	0.60	0.48	-0.25	4 (1)	0.53	0.70	0.24
A4JW7	3 (0)	0.41	0.46	0.11	3 (0)	0.75	0.61	-0.23	5 (4)	0.76	0.69	-0.10
9VSH	4 (0)	0.35	0.40	0.14	5 (1)	0.60	0.60	0.00	4 (1)	0.53	0.62	0.14
7PJQ	2 (0)	0.13	0.20	0.35	2 (0)	0.47	0.37	-0.29	2 (0)	0.12	0.11	-0.03
BWF1	4 (0)	0.57	0.63	0.09	4 (0)	0.28	0.61	0.55	4 (0)	0.59	0.64	0.08
3Q19	5 (1)	0.35	0.65	0.46	3 (0)	0.53	0.62	0.16	3 (0)	0.47	0.62	0.24
6U3A	7 (3)	0.48	0.74	0.36	5 (0)	0.37	0.80	0.54	4 (0)	0.24	0.22	-0.06
**Mean**	**4.58 (1.00)**	**0.45**	**0.58**	**0.58**	**4.25 (0.67)**	**0.55**	**0.63**	**0.09**	**3.58 (0.92)**	**0.58**	**0.58**	**0.58**
**SE**	**0.50 (0.37)**	**0.05**	**0.05**	**0.05**	**0.41 (0.36)**	**0.04**	**0.03**	**0.08**	**0.34 (0.38)**	**0.05**	**0.05**	**0.05**
Selfing populations												
Locus	Presolana (*T*_*m*_ = 0^a^)				Val de Roya 1 (*T*_*m*_ = 0.14)				Val de Roya 2 (*T*_*m*_ = 0.13)			
	*N*_A_	*H*_O_	*H*_E_	*F*_IS_	*N*_A_	*H*_O_	*H*_E_	*F*_IS_	*N*_A_	*H*_O_	*H*_E_	*F*_IS_
DJ5E	2 (1)	0.00	0.20	1.00	2 (0)	0.07	0.40	0.82	1 (0)	0.00	0.00	-
DEET	2 (1)	0.11	0.11	0.00	2 (0)	0.00	0.44	1.00	2 (1)	0.00	1.00	1.00
4MDH	1 (0)	0.00	0.00	-	1 (0)	0.00	0.00	-	1 (0)	0.00	0.00	-
A1T8T	1 (0)	0.00	0.00	-	1 (0)	0.00	0.00	-	1 (0)	0.00	0.00	-
5GTC	2 (1)	0.00	0.33	1.00	3 (0)	0.00	0.27	1.00	1 (0)	0.00	0.00	-
A93Q	2 (0)	0.00	0.18	1.00	1 (0)	0.00	0.00	-	2 (0)	0.00	0.67	1.00
A4JW7	4 (2)	0.30	0.44	0.33	2 (0)	0.07	0.47	0.85	2 (0)	0.25	0.25	0.00
9VSH	2 (1)	0.00	0.20	1.00	2 (0)	0.00	0.26	1.00	2 (0)	0.25	0.25	0.00
7PJQ	1 (0)	0.00	0.00	-	1 (0)	0.00	0.00	-	2 (0)	0.00	0.50	1.00
BWF1	2 (0)	0.36	0.31	-0.18	2 (0)	0.14	0.43	0.67	2 (0)	0.50	0.42	-0.20
3Q19	2 (0)	0.00	0.22	1.00	1 (0)	0.00	0.00	-	1 (0)	0.00	0.00	-
6U3A	3 (0)	0.09	0.41	0.78	3 (0)	0.00	0.27	1.00	2 (0)	0.00	0.67	1.00
**Mean**	**2.00 (0.50)**	**0.07**	**0.20**	**0.66**	**1.75 (0.00)**	**0.02**	**0.21**	**0.91**	**1.58 (0.08)**	**0.08**	**0.31**	**0.58**
**SE**	**0.85 (0.19)**	**0.03**	**0.04**	**0.16**	**0.22 (0.00)**	**0.01**	**0.06**	**0.05**	**0.15 (0.08)**	**0.05**	**0.10**	**0.05**

Number of alleles (*N*_*A*_) with number of private alleles in parentheses, observed heterozygosity (*H*_*O*_), expected heterozygosity (*H*_*E*_), and inbreeding coefficient (*F*_*IS*_). Classification was based on previous estimates of outcrossing rates *T*_*m*_ [34] and are indicated for each population. Multilocus means and standard errors (SE) are given for outcrossing and selfing populations.

^a^ This estimate was due to the complete absence of allozyme variation (cf. Tedder *et al*. 2011 for details).

## Discussion

### Pronounced changes towards the selfing syndrome in selfing populations of Arabis alpina

Selfing has evolved repeatedly throughout the angiosperms and is generally associated with changes in floral traits towards a selfing syndrome [51–53], among others characterised by smaller flowers, reduced herkogamy and reduced pollen: ovule ratios [18]. Our morphometric comparison of flowers from three outcrossing and three selfing populations of *Arabis alpina* revealed pronounced differences, with selfing populations had a morphology closer to a typical selfing syndrome (Figs 3 and 4). Such floral differences are commonly observed among closely related species with contrasting mating systems (e.g., outcrossing *Ipomoea cordatotriloba* and selfing *I*. *lacunosa*, [23]; outcrossing *Capsella grandiflora* and selfing *C*. *rubella* [16]), but can also occur intraspecifically. Our detailed description of floral syndromes (i.e., not only including flower size, but also measures of herkogamy and of pollen and ovule production) in relation to outcrossing and selfing in *A*. *alpina* adds to the limited number of studies that have documented intraspecific floral changes in relation to mating system variation (e.g., *Arenaria uniflora* [54]; *Leavenworthia alabamica* [55,56]; *Camissoniopsis cheiranthifolia* [57]; *Abronia umbellata* [58]).

### Size reduction and relative female and male investment

The observed floral morphological changes in the selfing populations were mainly attributable to an overall reduction in size of the flowers. The 2.5 fold reduction in petal area is not as strong as observed among the species *Capsella grandiflora* and *C*. *rubella*, in which the latter experienced a 6.5 fold reduction [24]. However, the size reduction appeared to be of a comparable magnitude with those observed within other species, such as *Arenaria uniflora* (3–5 fold reduced petal area [54]), *Camissoniopsis cheiranthifolia* (up to 2 fold reduction in petal length [57]) and *Abronia umbellata* (2–3 fold reduction in floral tube length [58]). Such size reductions are likely to influence pollinator visitation and florivory. In *Raphanus raphanistrum* for example, syrphid flies preferentially visited larger flowers [59] and in *Penstemon digitalis* pollinators exerted positive selection on flower size [60]). Selection on flower size by pollinators tends to be larger than by florivores [60], but there are examples of selection by florivores. In *Cistus ladanifer*, for example, florivores selectively fed on larger flowers [21], suggesting that trade-offs between pollinator attraction and herbivore preference may exist. The relative geographical proximity of large- (outcrossing) and small-flowered (selfing) populations offer exciting opportunities to experimentally address the role of pollinators and florivores as potential drivers of changes towards the selfing syndrome.

Pistil length did not differ between outcrossers and selfers (Fig 3 and Table 2) and formed an exception to the pattern of a general size reduction of floral traits. Since the ovule number also did not differ between mating systems (Fig 4 and Table 2), we propose that selection has favoured a size reduction of floral organs in general, with the exception of pistil length. Ovary lengths showed a similar pattern as pistil lengths (S1 Table). Possibly, ovary length and pistil length are intrinsically linked in *A*. *alpina*, and reducing ovary size (and thereby overall pistil length) would have provided a selective disadvantage because smaller ovaries may accommodate fewer ovules. Although further work is needed to test this hypothesis, our evidence of de-coupling of the ovary/pistil length from other floral size traits suggests that the female flower organs have a different genetic basis from other flower organs.

Theory predicts that selfing lineages need fewer pollen grains per ovule to guarantee optimal fertilisation, and—assuming a cost associated with pollen production—that selection should therefore lead to a reduction of the pollen production and specifically the pollen to ovule ratio [61]. In accordance with these expectations, we observed a reduction in pollen production (Fig 4 and Table 2), which resulted in a change in pollen to ovule ratio from an average of 2206 in outcrossing populations to 627 in selfing populations. It has been proposed that pollen to ovule ratios can be used as an indicator for mating system [62]. Although this has been debated [63], the pollen to ovule ratio has proven to be a fairly accurate predictor of mating system in the Brassicaceae [64]. Our estimates confirm this. For the outcrossing populations our estimates are within the range reported for allogamous Brassicaceae (29 species: 1100–38000, mean 9086 [64]), and our estimates for the selfing populations fall within the range reported for autogamous Brassicaceae (37 species, 21–3220, mean 646 [64]). The reduction in pollen to ovule ratios is normally due to a reduction in male investment (i.e., pollen production), although increases in ovule numbers in selfing lineages have also been observed [18,24]. Our data only revealed a marginal increase in pollen size (S1 Table), and so we predict that the resources saved on pollen production (and on decreased flower size) have been reallocated to female fitness components. Since ovule number remained unchanged in the selfing populations (Fig 4 and Table 2), it remains to be investigated whether selfing *A*. *alpina* achieves increased female fitness through other traits such as an increased flower number per plant and/or increased seed size.

### Reduced herkogamy for the long stamens

The pattern of reduced herkogamy for the long stamens and increased herkogamy for the short stamens (Fig 3 and Table 2) is obviously tightly linked to pistil length. The observed differences in herkogamy between selfing and outcrossing populations may be a by-product of selection for reduced flower size (with a constant pistil length), rather than selection for increased selfing efficiency. Alternatively, the observed differences in herkogamy may be the direct result of selection for increased selfing efficiency, of which reduced flower size could either be a by-product (for example due to pleiotropic effects) or be targeted by separate selective forces. In systems without clear size reductions, reduced herkogamy tends to be correlated with a reduction in outcrossing rate, as demonstrated in various species, including *Nicotiana* [65], *Clarkia* [66], *Turnera* [67], *Mimulus* [68], *Aquilegia* [69], and *Datura* [70], although there are exceptions (e.g., *Narcissus* [71,72]). Future work is needed to test whether reduced herkogamy in selfing syndromes is a by-product of selection for reduced overall flower size or the other way around (pleiotropy hypothesis [18]), or whether both traits are under selection simultaneously.

### An open question: single or multiple origins of selfing and the selfing syndrome?

The inbreeding coefficients (*F*_IS_) based on microsatellite markers we report here for *A*. *alpina* (Table 3), confirm previous findings of outcrossing rates and *F*_IS_ for the same set of populations based on allozymes [33,34]. An interesting difference with this earlier work is that the selfing Presolana population, which had appeared to be invariable based on allozyme markers [33,34], was considerably more variable (but still clearly inbreeding) in our study based on microsatellite markers (Table 3).

A Bayesian clustering analysis identified two clusters, which segregated by mating system and area of origin (Fig 5). Population genetic studies with more dense sampling of the entire Alpine range, where populations are generally assumed to be predominantly selfing, have suggested the existence of at least two genetic clusters based on allozymes [33] and AFLP data revealed even more sub-structuring [73]. The existence of multiple population genetic clusters within the selfing range of *A*. *alpina* is equally compatible with a single transition to selfing and subsequent divergence, or multiple origins of selfing from different genetic backgrounds. Hence, the question whether selfing and the selfing syndrome evolved once or multiple times cannot be answered at the moment.

### Conclusion

In this paper, we documented that in *Arabis alpina* three selfing populations (high *F*_IS_, low outcrossing rates) are associated with a typical selfing syndrome floral morphology, with smaller flowers, reduced herkogamy and reduced pollen numbers compared to three outcrossing populations (low *F*_IS_, high outcrossing rates). It remains to be tested whether this is merely a local phenomenon, or whether the selfing syndrome is consistently associated with self-fertilisation throughout the entire range of *Arabis alpina*. Our results show that *A*. *alpina—*given the available molecular, genomic and population genetic data—is a promising model system to study the evolution of selfing and the selfing syndrome within a single species. Among others, this offers opportunities to address the selective processes that drive the evolution of the selfing syndrome, and the underlying genetic and transcriptomic changes.

## Supporting Information

S1 Dataset Zip archive of tab-delimited text files. Each text file contains a specific dataset: herkogamy, ovule, petal area, pollen, P:O ratios, microsatellites genotypes. Text files starting with “Variable_explanation_…” contain explanations of each of the variables contained in the data files.
(ZIP)

S1 Fig Diagnostics for evaluation of STRUCTURE simulations. (A) Optimal cluster (K) estimation based on the *ΔK* method (B) Relation between likelihood (L) and cluster number.
(DOCX)

S1 Table Linear mixed model analysis of the effect of Mating System on floral traits of *Arabis alpina*. Cases where estimates for selfing populations differ significantly from those of the outcrossing populations are indicated in bold.
(DOCX)
