# Supplementary material for: Evolution of the Selfing Syndrome in Arabis alpina (Brassicaceae)
Source: PLoS One. 2015 Jun 3;10(6):e0126618. doi: 10.1371/journal.pone.0126618 (PMC4454584; doi:10.1371/journal.pone.0126618)

**S1 Figure Diagnostics for evaluation of STRUCTURE simulations.** (A) Optimal cluster (K) estimation based on the ΔK method Evanno *et al*. (2005) (B) Relation between likelihood (L) and cluster number.


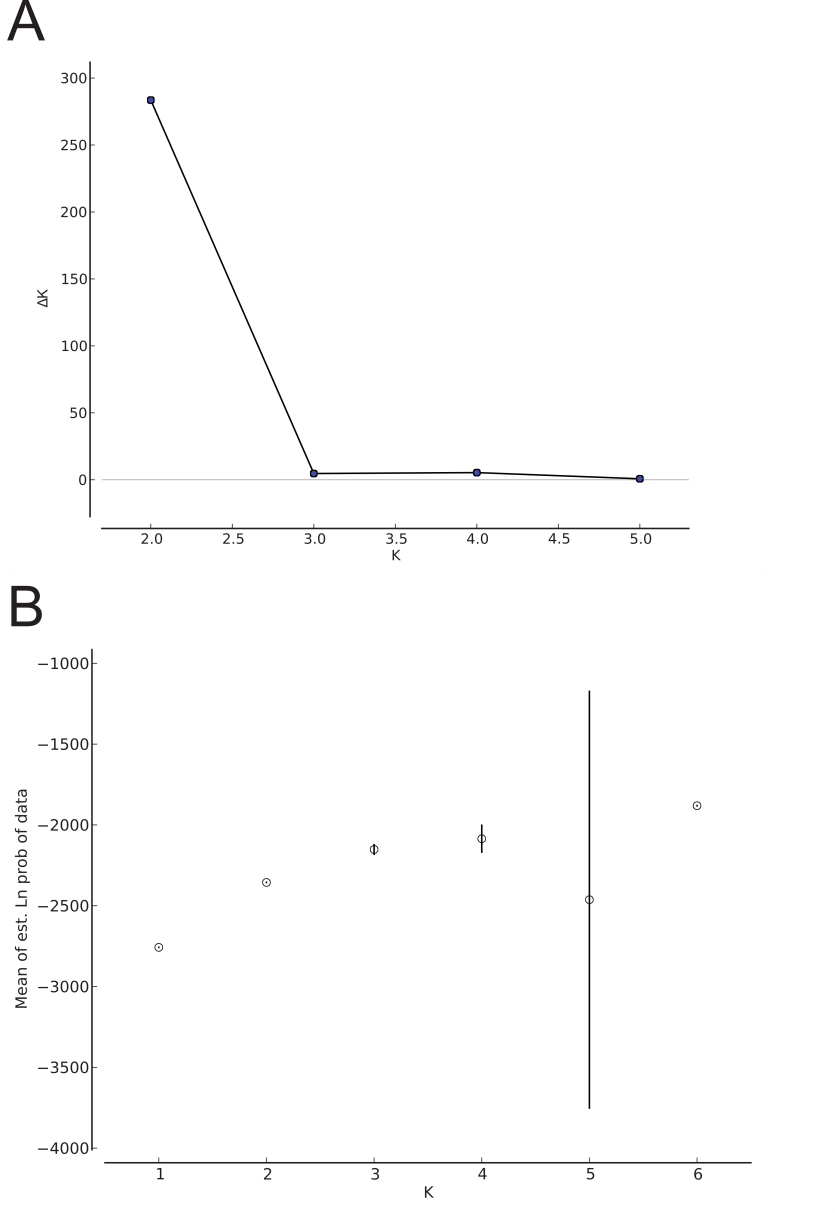

Supplement: S1 Fig — (A) Optimal cluster (K) estimation based on the ΔK method (B) Relation between likelihood (L) and cluster number. (DOCX) [file pone.0126618.s002.docx]
