# Supplementary material for: Evolution of the Selfing Syndrome in Arabis alpina (Brassicaceae)
Source: PLoS One. 2015 Jun 3;10(6):e0126618. doi: 10.1371/journal.pone.0126618 (PMC4454584; doi:10.1371/journal.pone.0126618)
Supplement: S1 Table — Cases where estimates for selfing populations differ significantly from those of the outcrossing populations are indicated in bold. (DOCX) [file pone.0126618.s003.docx]

**S1 Table. Linear mixed model analysis of the effect of Mating System on floral traits of *Arabis alpina*.** Cases where estimates for selfing populations differ significantly from those of the outcrossing populations are indicated in bold.

| **Trait (unit of measurement)** | **Transformation for analysis** | **Mean outcrossing (untransformed)** | **Mean selfing (untransformed)** | **Model estimate (transformed) of difference between outcrossing and selfing means^a^** | **t-value** | **P (df=4) ^b^** |
| --- | --- | --- | --- | --- | --- | --- |
| Dissection Index (mm/mm) | - | 5.92 | 5.94 | +0.03 | 0.31 | 0.771 |
| **Long stamen length (mm)** | **-** | **8.07** | **5.65** | **-2.41** | **-9.00** | **<0.001** |
| **Short stamen length (mm)** | **-** | **5.58** | **3.32** | **-2.27** | **-12.1** | **<0.001** |
| **Angle short stamens (degrees)** | **log_e_** | **16.9** | **25.3** | **+0.39** | **4.75** | **0.009** |
| **Angle long stamens (degrees)** | **Square root** | **2.90** | **5.81** | **+0.66** | **3.75** | **0.02** |
| **Pollen size  (μm)** | **-** | **19.77** | **20.84** | **+1.04** | **4.14** | **0.0144** |
| Ovary length  (mm) | **-** | 3.47 | 3.05 | -0.48 | -2.09 | 0.105 |

^a^ Model Fixed part: Mating system; Random part: Population and Plant_ID, analysed with the *lme* function in the *nlme* package in R (R-Core-Team, 2013)

^b^ df: degrees of freedom. For testing differences between mating system df=4 because there were three outcrossing and three selfing populations
